# N‐ethyl‐N‐nitrosourea–Induced Adaptor Protein 2 Sigma Subunit 1 (*Ap2s1*) Mutations Establish *Ap2s1* Loss‐of‐Function Mice

**DOI:** 10.1002/jbm4.10001

**Published:** 2017-05-02

**Authors:** Caroline M Gorvin, Angela Rogers, Michelle Stewart, Anju Paudyal, Tertius A Hough, Lydia Teboul, Sara Wells, Steve DM Brown, Roger D Cox, Rajesh V Thakker

**Affiliations:** ^1^ Academic Endocrine Unit Radcliffe Department of Medicine University of Oxford, Oxford Centre for Diabetes, Endocrinology, and Metabolism (OCDEM), Churchill Hospital Oxford UK; ^2^ Mary Lyon Centre and Mammalian Genetics Unit Medical Research Council Harwell Institute, Harwell Campus Oxfordshire UK

**Keywords:** DISORDERS OF CALCIUM/PHOSPHATE METABOLISM, PTH/VIT D/FGF23, PARATHYROID‐RELATED DISORDERS, CELL/TISSUE SIGNALING – ENDOCRINE PATHWAYS, ANIMAL MODELS – GENETIC ANIMAL MODELS

## Abstract

The adaptor protein‐2 sigma subunit (AP2σ), encoded by *AP2S1*, forms a heterotetrameric complex, with AP2α, AP2β, and AP2μ subunits, that is pivotal for clathrin‐mediated endocytosis, and AP2σ loss‐of‐function mutations impair internalization of the calcium‐sensing receptor (CaSR), a G‐protein–coupled receptor, and cause familial hypocalciuric hypercalcemia type‐3 (FHH3). Mice with AP2σ mutations that would facilitate investigations of the in vivo role of AP2σ, are not available, and we therefore embarked on establishing such mice. We screened >10,000 mice treated with the mutagen N‐ethyl‐N‐nitrosourea (ENU) for *Ap2s1* mutations and identified 5 *Ap2s1* variants, comprising 2 missense (Tyr20Asn and Ile123Asn) and 3 intronic base substitutions, one of which altered the invariant donor splice site dinucleotide *gt* to *gc*. Three‐dimensional modeling and cellular expression of the missense *Ap2s1* variants did not reveal them to alter AP2σ structure or CaSR‐mediated signaling, but investigation of the donor splice site variant revealed it to result in an in‐frame deletion of 17 evolutionarily conserved amino acids (del17) that formed part of the AP2σ α1‐helix, α1‐β3 loop, and β3 strand. Heterozygous mutant mice (*Ap2s1^+/del17^*) were therefore established, and these had AP2σ haplosufficiency but were viable with normal appearance and growth. *Ap2s1^+/del17^* mice, when compared with *Ap2s1^+/+^* mice, also had normal plasma concentrations of calcium, phosphate, magnesium, creatinine, urea, sodium, potassium, and alkaline phosphatase activity; normal urinary fractional excretion of calcium, phosphate, sodium, and potassium; and normal plasma parathyroid hormone (PTH) and 1,25‐dihydroxyvitamin D (1,25(OH)_2_) concentrations. However, homozygous *Ap2s1^del17^* ^*/del*^ *^17^* mice were non‐viable and died between embryonic days 3.5 and 9.5 (E3.5–9.5), thereby indicating that AP2σ likely has important roles at the embryonic patterning stages and organogenesis of the heart, thyroid, liver, gut, lungs, pancreas, and neural systems. Thus, our studies have established a mutant mouse model that is haplosufficient for AP2σ. © 2017 The Authors. *JBMR Plus* is published by Wiley Periodicals, Inc. on behalf of the American Society for Bone and Mineral Research.

## Introduction

Familial hypocalciuric hypercalcemia (FHH) is an autosomal dominant disorder characterized by lifelong elevations in serum calcium concentrations in association with normal or mildly elevated serum parathyroid hormone (PTH) concentrations and low urinary calcium excretion (urinary calcium clearance to creatinine clearance ratio <0.01).^1^, ^2^ FHH is a genetically heterogeneous disorder with three recognized forms referred to as FHH types 1–3 (FHH1–3).^3^ FHH1 (OMIM #145980) is caused by heterozygous loss‐of‐function mutations of the calcium‐sensing receptor (CaSR), which is a G‐protein–coupled receptor (GPCR), that preferentially activates the G‐protein subunit α_q/11_ (Gα_q/11_) family, with resulting increases in phospholipase C (PLC) activity and elevations of inositol 1,4,5‐trisphosphate (IP_3_) that lead to rapid increases in intracellular calcium (Ca^2+^_i_) concentrations.^4^, ^5^ Initiation of these CaSR‐signaling pathways leads to reduced circulating PTH and increased urinary calcium concentrations.^5^ Loss‐of‐function mutations of the Gα_11_ subunit, encoded by the *GNA11* gene, cause FHH2 (OMIM #145981),^6^, ^7^ whereas FHH3 (OMIM #600740) is caused by heterozygous loss‐of‐function mutations of the adaptor protein‐2 σ subunit (AP2σ), encoded by the *AP2S1* gene.^8^ AP2σ is the smallest subunit of the heterotetrameric AP2 protein, which comprises two large subunits, α and β, and two smaller subunits μ and σ.^9^ AP2 has a fundamental role in clathrin‐mediated endocytosis of GPCRs such as the CaSR,^10^ and to facilitate this, the AP2 α and β subunits have large appendages that bind to the clathrin coat protein, accessory proteins (eg, β‐arrestin), and plasma membrane phospholipids, thereby facilitating their roles as endocytic hubs.^11^ The AP2μ and σ subunits are involved in binding to motifs of cargo proteins with the μ subunit recognizing tyrosine‐based motifs and the σ subunit dileucine‐based motifs.^9^ All of the FHH3‐associated mutations reported to date involve the AP2σ Arg15 residue, and each of the 3 different missense mutations (Arg15Cys, Arg15His, and Arg15Leu)^8^ are postulated to disrupt the polar contacts between the AP2σ Arg15 residue and the dileucine motif in the cytoplasmic tail of the CaSR to target it for endocytosis.^8^, ^9^, ^12^

Mouse models harboring deleted alleles for CaSR, Gα_11_, and Gα_q_ have been established, and their investigations have provided important insights into the pathophysiological mechanisms of these disorders of calcium metabolism. Thus, mice deleted for one CaSR allele (ie, heterozygous knockout *CaSR^+/−^* mice) have been shown to develop modest hypercalcemia with relative hypocalciuria, and inappropriately elevated serum PTH, consistent with features of FHH1 in patients.^13^ In addition, homozygous knockout mice, ie, *CaSR^‐/‐^*, have been demonstrated to develop early onset of severe hypercalcemia in association with increased serum PTH concentrations, parathyroid hyperplasia, and bone demineralization that resulted in death by age 30 days.^13^, ^14^ These findings in *CaSR^‐/‐^* mice are representative of the features found in patients with neonatal severe primary hyperparathyroidism (NSHPT), who usually harbor homozygous or compound heterozygous CaSR mutations.^3^, ^15^, ^16^, ^17^ Moreover, studies of *CaSR^‐/‐^*/*Pth^‐/‐^* mice, which did not have increased neonatal lethality or skeletal abnormalities, have shown that CaSR has PTH‐independent roles in calcium homeostasis.^18^ These PTH‐independent roles of the CaSR, which are of importance in defending against hypercalcemia and in maintaining normocalcemia, include CaSR‐stimulated calcitonin secretion and increased renal calcium excretion.^19^ Mice with germline deletions of Gα_11_ and Gα_q_ alleles have also been generated, and *Gna11* ^‐/‐^ and *Gnaq* ^‐/‐^ have been reported to not develop abnormalities of calcium homeostasis.^20^ However, parathyroid‐specific deletions of both copies of Gα_11_ and Gα_q_ did result in hypercalcemia but without hypocalciuria, thereby indicating critical roles for Gα_11_ and Gα_q_ in mediating inhibition of PTH secretion by extracellular calcium [Ca^2+^_e_].^20^ To date, mice deleted for *Ap2s1* or harboring AP2σ loss‐of‐function mutations have not been reported, and we therefore pursued studies to establish such mice by screening a DNA archive of >10,000 samples from male mice that had mutations induced by treating them with N‐ethyl‐N‐nitrosourea (ENU).

ENU is an alkylating agent that primarily introduces point mutations via transfer of the ENU alkyl group to the DNA base followed by mispairing and subsequent base pair substitution during the next round of DNA replication.^21^, ^22^ ENU mutagenesis programs utilize two complementary approaches that are phenotype‐driven and genotype‐driven screens. In phenotype‐driven screens, offspring of mutagenized mice are assessed for abnormalities in a hypothesis‐generating strategy, which may elucidate new genes, pathways, and mechanisms for disease phenotypes.^21^, ^22^ Genotype‐driven screens in which mutations in the gene of interest are sought are hypothesis‐driven and are feasible by available parallel archives of tissue‐DNA and sperm samples from mutagenized male mice.^21^, ^22^ The archived tissue‐DNA samples from the mutagenized male mice are used to search for the mutations in the gene of interest, and once these mutations are found, then a sperm sample from the male mouse with the mutation is used for in vitro fertilization (IVF) of normal female mice to establish progeny with the mutation.^21^, ^22^ The probability of finding three or more variant alleles in an archive of tissue‐DNA samples from >5000 ENU‐mutagenized mice is >90%.^21^ We sought for such ENU‐induced *Ap2s1* variants in tissue‐DNA samples from >10,000 mice treated with ENU and identified five variants that were further appropriately assessed for their in vitro and in vivo effects on CaSR signaling and calcium homeostasis, respectively.

## Materials and Methods

### Animals

Animal studies were carried out using guidelines issued by the UK Medical Research Council in Responsibility in Use of Animals for Medical Research (July 1993) and UK Home Office project license numbers (PPL30/2433 and PPL30/3271). ENU‐treated G0 C57BL/6J male mice were mated to C3H/HeH (C3H) to produce G1 progeny, and tissue‐DNA samples from >10,000 G1 ENU mutagenized male mice, together with their sperm, were archived, as previously reported.^22^ The tissue‐DNA samples were used to identify *Ap2s1* variants by melt curve analysis of PCR products utilizing a Lightscanner and gene‐specific primers (BioFire Diagnostics, Inc., Salt Lake City, UT, USA), and sperm from mice with *Ap2s1* variants was used for IVF to generate G2 progeny on a C3H background strain, as reported.^23^ Heterozygous mutant male and female mice were intercrossed to generate homozygous mice, which were studied with their heterozygous and wild‐type littermates. Mice were fed on a standard diet (Rat and Mouse number 3, Special Diet Services, Essex, UK) that contained 1.15% calcium, 0.58% phosphate, and 4089 IU/kg of vitamin D, and provided with water *ad libitum*.^24^

### DNA sequence analysis

Genomic DNA was isolated from auricular biopsies using DNA extraction buffer (10 mM NaCl, 20 mM Tris‐HCl, pH8.0, 1 mM EDTA, 10% sodium dodecyl sulfate [SDS]) and Proteinase K solution (ThermoFisher, Carlsbad, CA, USA).^23^, ^24^ Genomic DNA was used with *Ap2s1* gene‐specific primers (Sigma‐Aldrich, Gillingham, UK) (Supplemental Table S1) to perform PCR amplification, followed by dideoxynucleotide sequencing using the BigDye Terminator v3.1 Cycle Sequencing Kit and an automated detection system (ABI 3730 Automated capillary sequencer, ThermoFisher), as previously reported.^23^, ^24^, ^25^ Polyphen‐2 (http://genetics.bwh.harvard.edu/pph2/)^26^ and MutationTasting (http://www.mutationtaster.org/)^27^ were used to predict the effects of missense mutations.

### Protein sequence alignment and three‐dimensional modeling

Protein sequences of AP2σ were aligned using ClustalOmega (http://www.ebi.ac.uk/Tools/msa/clustalo/).^28^ PyMOL Molecular Graphics System (Version 1.8, Schrödinger, LLC, Portland OR, USA) was used to model the effects of the AP2σ variants, using the reported three‐dimensional structure of AP2σ.^8^, ^9^

### RNA extraction, reverse transcription‐PCR (RT‐PCR), and quantitative PCR (qRT‐PCR)

Total RNA was isolated from auricular biopsies using Trizol reagent (ThermoFisher), as described.^29^ cDNA was prepared from 1 μg of RNA using the QuantiTect Reverse Transcription Kit (Qiagen, Manchester, UK). RT‐PCR was performed using gene‐specific primers encompassing *Ap2s1* exons 1–3 (Supplemental Table S1). PCR products were gel purified using the Geneclean Kit (MP Biomedicals, Santa Ana, CA, USA) and cloned into the pCR‐BluntII‐TOPO vector (Life Technologies, Carlsbad, CA, USA), and the DNA sequences of the inserts determined using vector‐specific primers (Supplemental Table S1), as described.^25^ qRT‐PCR reactions were performed using the QuantiTect SYBR Green Kit (Qiagen) in four independent samples utilizing a Rotorgene 5 (Qiagen), as described previously.^30^ All qRT‐PCR test samples were normalized to the geometric mean of three housekeeper genes (cyclin D1 [*Ccnd1*], glyceraldehyde 3‐phosphate dehydrogenase [*Gapdh*], and phosphoglycerate kinase 1 [*Pgk1*]), as described previously).^30^ Threshold cycle (C_T_) values were obtained from the start of the log phase on Rotorgene Q Series Software and C_T_ values analyzed in Microsoft Excel 97–2010 using the Pfaffl method.^30^ Data for mutant mice were expressed relative to wild‐type mice, expressed as 1. For confirmation of the effects of splicing, cDNA was PCR amplified using gene‐specific primers (Supplemental Table S1), utilizing methods previously described (Sigma‐Aldrich).^30^ Predicted effect of mutations in introns was assessed using the Alternative Splicing Site Predictor (wangcomputing.com/asp/).^31^

### Western blot analysis

Whole kidney lysates from wild‐type and mutant littermates were prepared using modified RIPA lysis buffer (50 mM Tris HCl, pH7.4, 150 mM NaCl, 1% (vol/vol) Igepal CA630, 0.5% sodium deoxycholate, 0.1% SDS, 1 mM sodium orthovanadate, Roche [Mannheim, Germany] protease inhibitors), as previously described.^25^ Lysates of flow cytometry cells were prepared using NP40 lysis buffer (50 mM Tris HCl, pH7.4, 1 mM EDTA, 150 mM NaCl, protease inhibitors), as described.^25^ The lysates were resuspended in Laemmli buffer; boiled and separated on 10% SDS polyacrylamide electrophoresis gels; transferred to polyvinylidene difluoride membranes (Amersham Life Sciences, Buckinghamshire, UK), which were blocked in 5% BSA/TBS‐T; appropriately probed using antibodies for an AP2σ, AP2μ, AP2β, AP2α, calnexin, and/or CaSR; and visualized using Immuno‐Star WesternC Kit (BioRad, Hercules, CA, USA) on a BioRad Chemidoc XRS+ system and densitometry analysis was performed using ImageJ, as previously described.^25^ Statistical analyses were performed by 2‐way ANOVA.

### Cell culture and transfection for flow cytometry analysis

Wild‐type and mutant pBI‐CMV4‐*AP2S1* expression constructs were generated, as described^12^ and transiently transfected into human embryonic kidney (HEK)‐293 cells stably expressing CaSR (HEK‐CaSR)^7^, ^8^ using Lipofectamine 2000 (Life Technologies). The bidirectional pBI‐CMV4 cloning vector was used because it facilitated the co‐expression of AP2σ and red fluorescent protein (RFP).^12^ Site‐directed mutagenesis was used to generate the mutant *AP2S1* constructs using the Quikchange Lightning Site‐Directed Mutagenesis Kit (Agilent Technologies, Santa Clara, CA, USA) and gene‐specific primers (Sigma‐Aldrich), as described.^25^ Cells were maintained in Dulbecco's Modified Eagle Medium (DMEM)‐Glutamax media (ThermoFisher) with 10% fetal bovine serum (Gibco) and 400 μg/mL geneticin (ThermoFisher) at 37 °C, 5% CO_2_. Transfection was confirmed by Western blot analyses and by visualizing RFP fluorescence using an Eclipse E400 fluorescence microscope with a Y‐FL Epifluorescence attachment and a triband 4,6‐diamidino‐2‐phenylindole‐FITC‐Rhodamine filter, and images taken using a DXM1200C digital camera and NIS Elements software (Nikon), as described.^12^

### Intracellular calcium measurements by flow cytometry analysis

The Ca^2+^_i_ responses of HEK293‐CaSR cells expressing wild‐type variants or mutant AP2σ proteins were assessed by a flow cytometry‐based assay, as reported.^6^, ^7^, ^12^ In brief, HEK293‐CaSR cells were cultured in T75 flasks and transiently transfected 24 hours later with 8 μg DNA.^(6)^ Forty‐eight hours after transfection, cells were detached, resuspended in calcium (Ca^2+^)‐ and magnesium (Mg^2+^)‐free Hanks’ buffered saline solution (HBSS), and loaded with 1 μg/mL Indo‐1‐acetoxymethylester (Indo‐1‐AM) for 1 hour at 37 °C. After removal of free dye, cells were resuspended in Ca^2+^‐ and Mg^2+^‐free HBSS and maintained at 37°C. Transfected cells, in suspension, were stimulated by sequentially adding Ca^2+^ to the Ca^2+^‐ and Mg^2+^‐free HBSS to increase the extracellular calcium concentration ([Ca^2+^_e_]) in a stepwise manner from 0 to 15 mM and then analyzed on a MoFlo modular flow cytometer (Beckman Coulter, High Wycombe, UK) by simultaneous measurements of RFP expression, Ca^2+^_i_‐bound Indo‐1AM, and free Indo‐1AM (ie, not bound to Ca^2+^_i_), using a JDSU Xcyte UV laser (Coherent Radiation, Santa Clara, CA, USA), on each cell at each [Ca^2+^_e_], as described.^7^ The peak mean fluorescence ratio of the Ca^2+^_i_ transient response after each individual stimulus was measured using Cytomation Summit software (Beckman Coulter) and expressed as normalized responses, as described.^7^ Nonlinear regression of concentration‐response curves was performed with GraphPad Prism (GraphPad, La Jolla, CA, USA) using the normalized response at each [Ca^2+^_e_] for each separate experiment for the determination of EC_50_ (ie, [Ca^2+^_e_] required for 50% of the maximal response). The maximal signaling response was measured as a fold‐change of the peak transient Ca^2+^_i_ response to the basal Ca^2+^_i_ response measured at 0 mM Ca^2+^_e_. The maximal signaling responses for variant and mutant AP2σ proteins were expressed as a percentage of the wild‐type AP2σ protein maximal signaling response. The mean EC_50_ obtained from 4 separate transfection experiments were used for statistical comparison by using the *F* test, and alterations in maximal signaling responses assessed using the Mann‐Whitney *U* test.

### Metabolic cage studies, plasma and urine biochemistry, and hormone analysis

Ten‐ to 12‐week‐old G2 mice were individually housed in metabolic cages (Techniplast, Kettering, UK) for 24 hours with free access to food and water.^32^ Mice were allowed to acclimatize to their environment over a 72‐hour period, as described,^32^ before collection of 24‐hour urine samples. Food and water intake was monitored, and mice were weighed before and after the study. Twenty‐four‐hour urine samples were collected in tubes containing sodium azide, and blood samples were collected from the lateral tail vein or the internal jugular vein into lithium heparin Microvette tubes (Sarstedt, Leicester, UK) after terminal anesthesia, as previously described.^24^ Urine and plasma analyses were performed using a Beckman Coulter AU680 analyzer, as reported.^24^ Plasma and urine were appropriately analyzed for sodium, potassium, total calcium, phosphate, magnesium, urea, creatinine, and alkaline phosphatase activity on a Beckman Coulter AU680 analyzer. Plasma calcium was adjusted for variations in albumin concentrations using the formula: (plasma calcium (mmol/L) − [(plasma albumin (g/L) − 30) × 0.02], as reported.^24^ Hormones were measured as follows: PTH using two‐site ELISA specific for mouse intact PTH (Immutopics, San Clemente, CA, USA), and 1,25‐dihydroxyvitamin D by a two‐step process involving purification by immunoextraction and quantification by enzyme immunoassay (Immunodiagnostic Systems, Baldan, UK), as described.^24^, ^33^ The fractional excretion of sodium, potassium, and calcium were calculated using the formula U_x_/P_x_*P_Cr_/U_Cr_, where U_x_ is the urinary concentration of the filtered substance (substance *x*) in mmol/L, P_x_ is the plasma concentration of substance *x* in mmol/L, U_Cr_ is the urinary concentration of creatinine in mmol/L, and P_Cr_ is the plasma concentration of creatinine in mmol/L.^24^ A Mann‐Whitney *U* test was used to compare differences between wild‐type and mutant littermates, with a value of *p* < 0.05 being considered significant for all analysis.

### Embryo analysis

Blastocysts at embryonic day 3.5 (E3.5) and embryos from E9.5 and E12.5 were collected by flushing (E3.5) or dissecting the uterine horns (E9.5 and E12.5) from pregnant females post‐mortem. Embryo age was confirmed by assessing the development of external structures as described.^34^, ^35^ Amniotic sacs were collected for genotyping as described.^34^

## Results

### Identification of five *Ap2s1* variants

**Figure 1:**
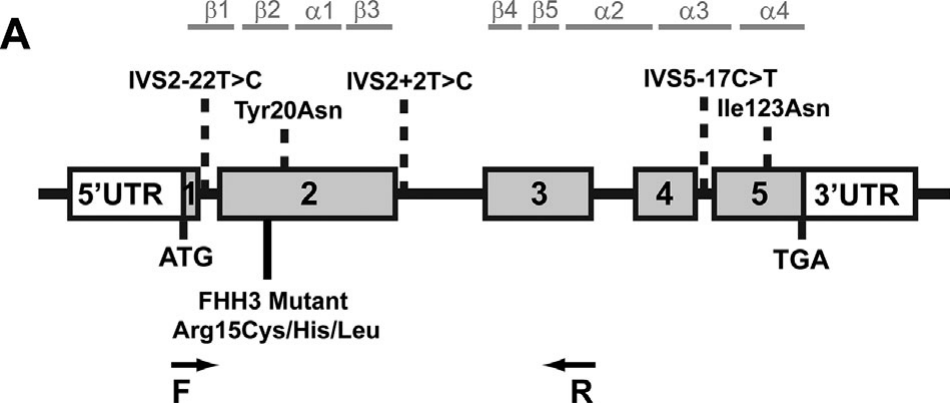
Identification of five variants of the *Ap2s1* gene induced by ENU in male mice. Schematic representation of the genomic organization of the mouse *Ap2s1* gene. The *Ap2s1* gene consists of five exons with the start (ATG) and stop (TGA) codons located in exons 1 and 5, respectively. The 5 ENU‐induced variants identified from screening tissue‐DNA samples from >10,000 male mice treated with ENU are highlighted by dotted lines above the exons and introns. The variants are two missense substitutions, Tyr20 and Ile123Asn, located in exons 2 and 5, respectively, and three intronic variants located in introns 1, 2, and 4. The locations of the FHH3‐associated mutations (Arg15Cys, Arg15His, and Arg15Leu) in humans are shown below the exons and introns. This Arg15 residue, which is located in exon 2, is conserved in mouse. The locations of the primers in exon 1 and 3 to investigate the effects of the intron variant located in intron 2 (IVS2+2T>C) are shown by arrows (F [forward] and R [reverse] primers).

Five *Ap2s1* variants comprising 2 within exons, which predicted missense substitutions Tyr20Asn and Ile123Asn, and 3 within introns were identified by screening the tissue‐DNA samples from >10,000 ENU‐mutagenized male mice for abnormalities involving the five exons and intron‐exon boundaries of the full‐length *Ap2s1* gene (Fig. 1). The effects of these variants were initially investigated by structural and in vitro functional studies that examined for alterations in CaSR signaling via Gα_q/11_ and increases in [Ca^2+^]_i_.

### Structural and in vitro signaling studies of the Tyr20Asn and Ile123Asn AP2σ variants

**Figure 2:**
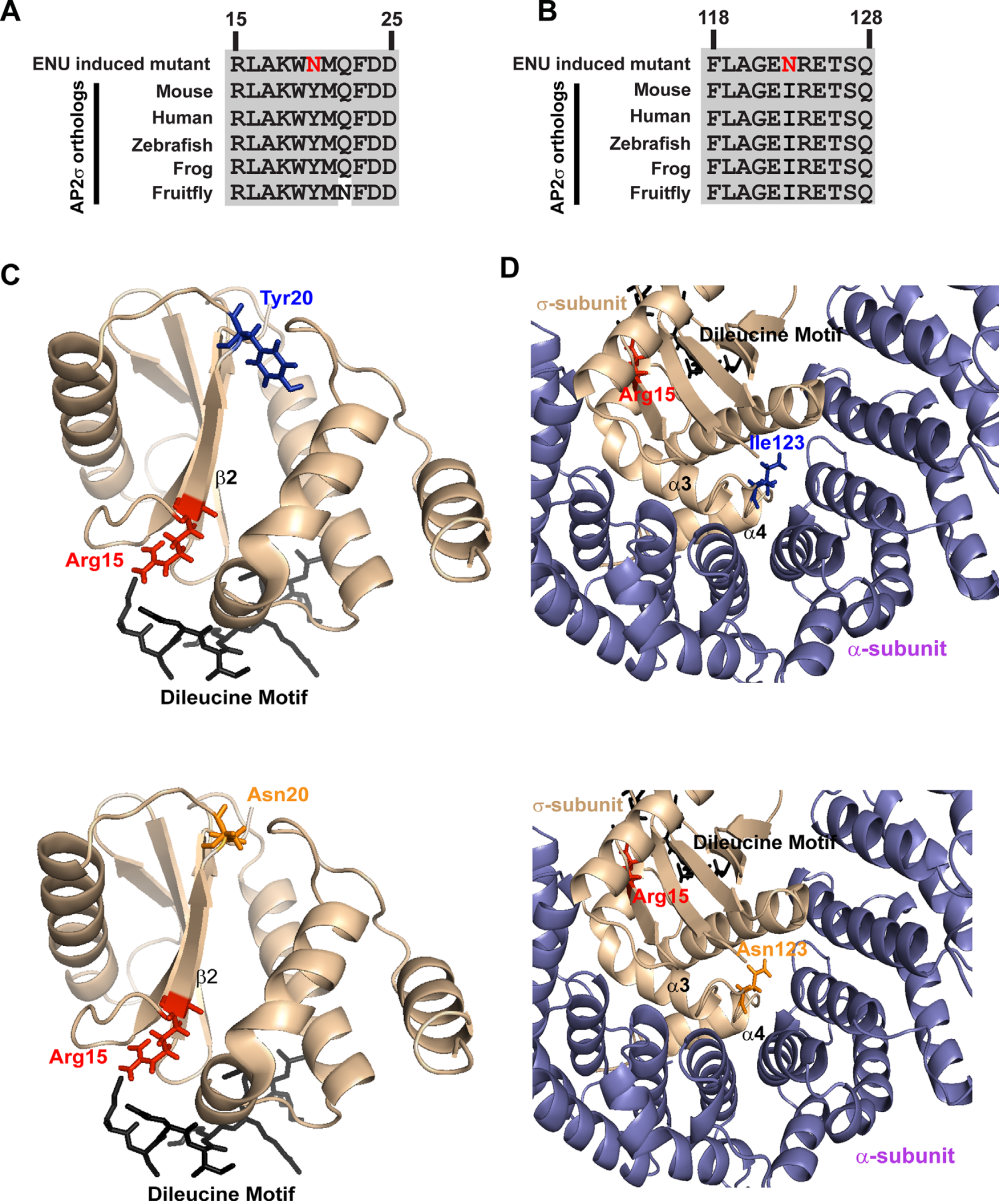
*Ap2s1* missense variants affect conserved residues. Multiple protein sequence alignment of residues (*A*) 15 to 25 and (*B*) 118 to 128 of AP2σ orthologs show positions of Tyr20 (Y20) and Ile123 (I123), respectively. The Y20 and I123 residues are evolutionarily conserved, thereby indicating that they may have important structure‐function roles in AP2σ. Conserved residues are shown in gray and the variants, both Asn (N), are shown in red. Homology model of the AP2σ protein (light brown) shows the predicted structural effects of (*C*) Tyr20Asn and (*D*) Ile123Asn. Wild‐type residues are shown in blue; mutant residues in orange. To date, FHH3 mutations involving only residue Arg15 (red) that lies within the second β‐strand of AP2σ have been identified. This Arg15 residue is predicted to bind to a putative dileucine motif of the CaSR (black). The Tyr20 residue lies distal to the Arg15 residue within β2 and on the opposite side of the dileucine binding region. The function of this AP2σ region containing Tyr20, which is not predicted to form polar contacts with other residues, is unknown. Importantly, the Asn variant does not disrupt or form new contacts, making it difficult to predict its effects. The Ile123 residue lies within a loop between the two terminal helices (α3–α4) of AP2σ, which lies close to the AP2α subunit. However, the Asn123 variant is not predicted to disrupt contacts with other AP2σ or AP2α residues, thereby making it difficult to predict its effects.

Of the two missense variants, the Tyr20Asn was located in exon 2 and was due to a T‐to‐A transversion, and the Ile123Asn was located in exon 5 and was also due to a T‐to‐A transversion (Fig. 1). Bioinformatic analysis using Polyphen‐2 and MutationTasting software^(26,27)^ predicted the AP2σ variants (Tyr20Asn and Ile123Asn) to be damaging and likely disease‐causing (Polyphen‐2 score 1, MutationTasting score 0.99 for both variants). In addition, the evolutionary conservation of the Tyr20 and Ile123 residues in AP2σ (Fig. 2 *A*, *B*) and absence of DNA sequence abnormalities that would alter the Tyr20 and Ile123 residues from >66,000 exomes (combined from the NHLBI‐ESP and ExAC cohorts)^36^ indicated that the Tyr20Asn, which is located 5 residues C‐terminal from the FHH3‐associated mutations at Arg15, and Ile123Asn variants could represent pathogenic mutations rather than benign polymorphisms. However, three‐dimensional modeling of the variants using the crystal structure of the AP2 heterotetramer in association with a dileucine motif of a cargo protein^9^ indicated that they were unlikely to alter the structure of AP2 or disrupt polar contacts with the dileucine motif (Fig. 2 *C*, *D*). Thus, the Tyr20 residue, which lies within the second β‐strand of AP2σ that is also the location of the FHH3‐associated Arg15 mutations, is situated away from the dileucine binding region and is also not predicted to form contacts with any other residues in AP2σ or in the AP2 complex (Fig. 2 *C*). The Ile123 residue, which is located within a loop structure between 2 α‐helices (α3–α4) that lie close to the AP2α subunit (Fig. 2 *D*), is also not predicted to contact residues in AP2σ or AP2α. Thus, the consequences of the Tyr20Asn and Ile123Asn variants are difficult to predict, and functional in vitro studies were undertaken to determine the effects on CaSR‐mediated signaling.

**Figure 3:**
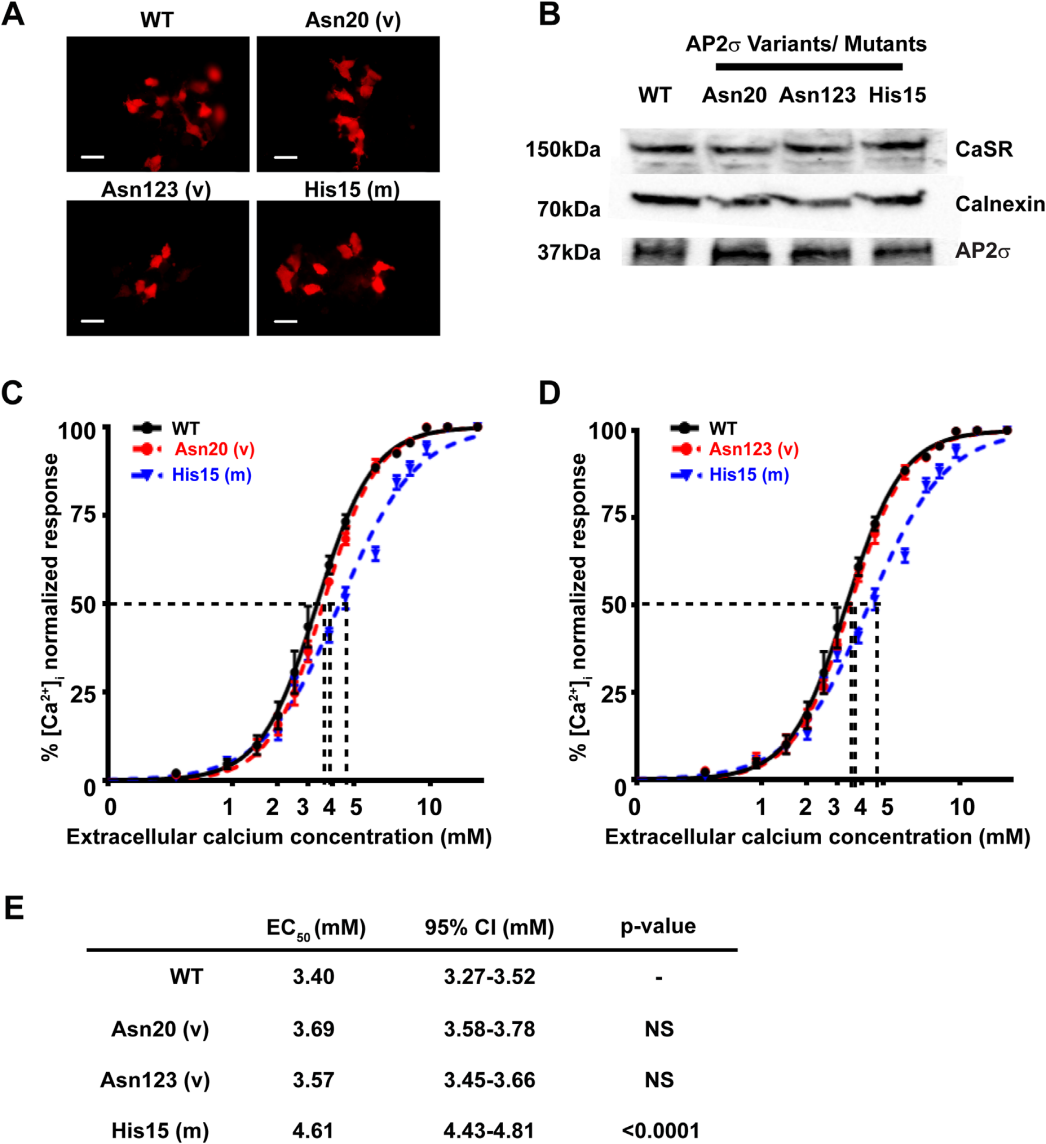
Intracellular calcium responses of the ENU‐induced AP2σ variants Tyr20Asn and Ile123Asn. (*A*) Fluorescence microscopy of HEK293 cells stably expressing CaSR (HEK‐CaSR) and transiently transfected with wild‐type (WT), ENU variants (v) Tyr20Asn (Asn20) or Ile123Asn (Asn123), or the FHH3‐associated Arg15His (His15) mutant (m) pBI‐CMV4‐*AP2S1* constructs. RFP expression in these cells indicates successful transfection and expression by these constructs. Scale bar = 10 μm. (*B*) Western blot analysis of lysates from HEK‐CaSR cells used for flow cytometry experiments. Transient transfection with WT, variant Asn20 and Asn123, or mutant His15 constructs resulted in similar levels of overexpression of AP2σ. Calnexin, a housekeeping protein, was used as a loading control. Ca^2+^_i_ response to changes in [Ca^2+^_e_] of HEK‐CaSR cells transfected with WT, (*C*) variant (v) Asn20, (*D*) variant Asn123 or mutant (m) His15 expression constructs. The Ca^2+^_i_ responses to changes in [Ca^2+^_e_] are expressed as a percentage of the maximum normalized responses and shown as the mean ± SEM of 4 independent transfections. The FHH3‐associated His15 AP2σ mutant led to a rightward shift in the concentration‐response curve, as previously reported,^(8)^ whereas the Asn20 and Asn123 variants were indistinguishable from WT. (*E*) Table showing the mean half‐maximal concentration (EC_50_) with 95% confidence intervals (CI) and *p* values.

CaSR‐mediated signaling was assessed using HEK‐CaSR cells that were transiently transfected with pBI‐CMV4 constructs that expressed the wild‐type or variant Asn20 and Asn123 AP2σ proteins, or FHH3‐associated Arg15His (His15) AP2σ mutant protein,^7^ and measuring their Ca^2+^_i_ responses to alterations in [Ca^2+^_e_]. Expression of the CaSR, AP2σ, and RFP was confirmed by fluorescence microscopy and/or Western blot analysis (Fig. 3 *A*, *B*). AP2σ expression was demonstrated to be similar in cells transiently transfected with wild‐type, variant AP2σ, and FHH3‐mutant AP2σ proteins (Fig. 3 *A*, *B*). The Ca^2+^_i_ responses in wild‐type, variant (Asn20 and Asn123), and FHH3‐mutant His15 AP2σ‐expressing cells were shown to increase in a dose‐dependent manner after stimulation with increasing concentrations of Ca^2+^_e_ (Fig. 3 *C*, *D*). Exposure to a significantly greater [Ca^2+^_e_] was required to achieve half‐maximal (EC_50_) Ca^2+^_i_ responses for cells expressing the FHH3‐mutant His15 (Fig. 3 *C*–*F*), as previously reported.^8^ However, EC_50_ values for Asn20 and Asn123 AP2σ variants were not significantly different from WT responses (Fig. 3 *C*–*E*). Thus, the FHH3‐associated His15 mutant‐expressing cells showed rightward shifts in the concentration‐response curves, with significantly elevated mean EC_50_ values (*p* < 0.0001) of 4.61 mM (95% confidence interval [CI] 4.43–4.81 mM), compared with 3.40 mM (95% CI 3.27–3.52 mM) for wild‐type–expressing cells, 3.69 mM (95% CI 3.58–3.78 mM) for Asn20–expressing cells, and 3.57 mM (95% CI 3.45–3.66 mM) for Asn123–expressing cells (Fig. 3 *E*). These results reveal that although the Asn20 and Asn123 variants are rare and involve evolutionarily conserved residues, they nevertheless do not affect the structure of AP2σ or CaSR‐mediated signaling. The mutant mice harboring the Asn20 and Asn123 variants were therefore not rederived for in vivo studies.

### Studies of 3 intronic *Ap2s1* variants on splicing and AP2σ structure

**Figure 4:**
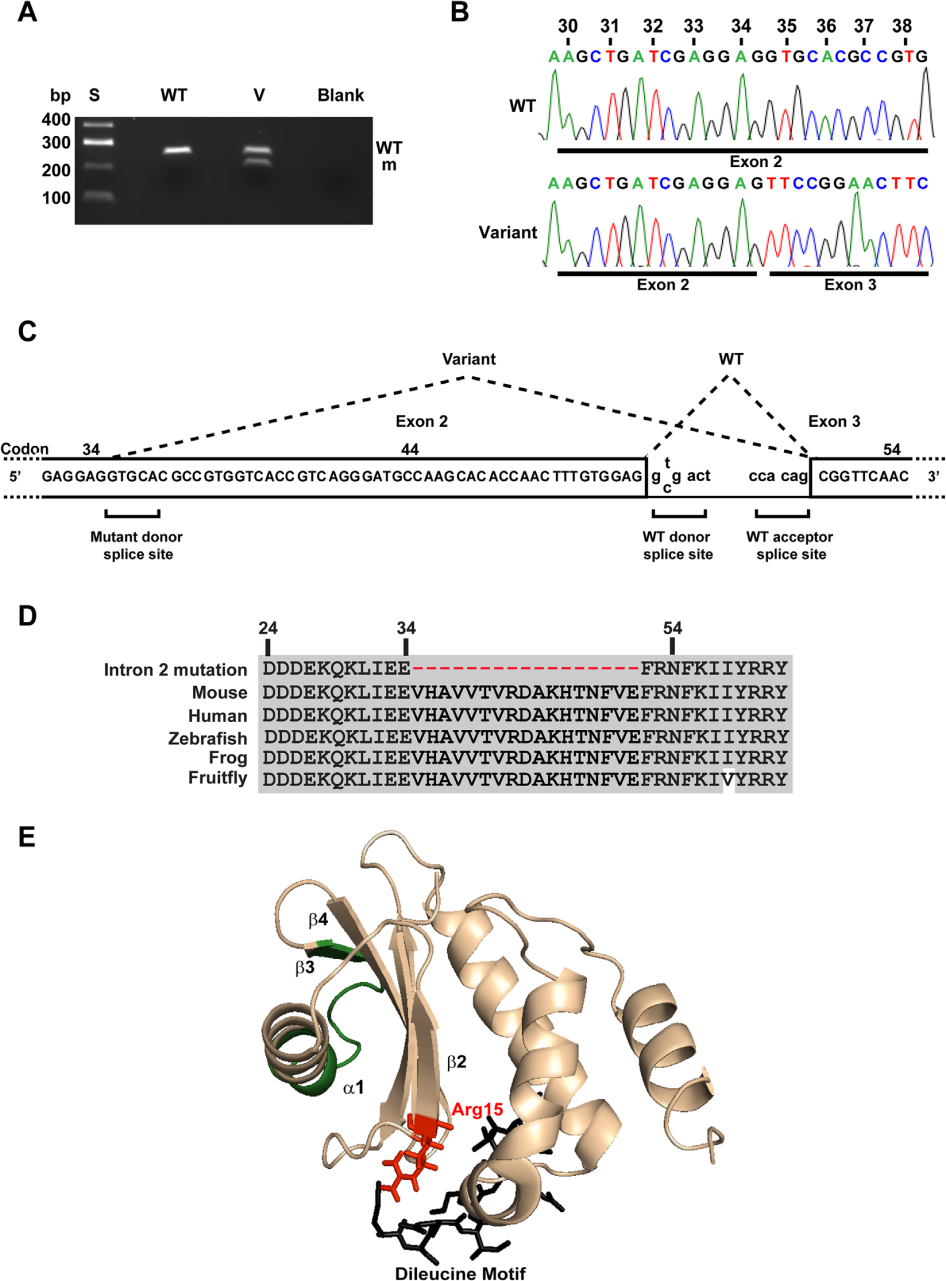
Intron 2 donor splice site variant (T>C) leads to abnormal mRNA splicing due to loss of donor splice site and utilization of a cryptic donor splice site in exon 2. (*A*) The effect of the intron 2 donor splice site variant T>C, which resulted in alteration of the invariant *gt* dinucleotide to *gc*, was assessed by RT‐PCR using primers located in *Ap2s1* exons 1 and 3 (Fig. 1), utilizing total RNA obtained from a wild‐type (WT) mouse and a mouse with the variant (v). Only one RT‐PCR product was obtained from the WT mouse at 267 bp, whereas two products were obtained from the heterozygous variant mouse, at 267 bp and 216 bp. S = size marker. (*B*) DNA sequence analysis of the variant heterozygous mouse revealed that the wild‐type product had full‐length exon 2, whereas the variant product lacked the last 51 bp of exon 2 that resulted in a loss of 17 amino acids (codons 35–51). This mutation, resulting in loss of 17 amino acids, was therefore designated del17 and the heterozygous mouse referred to as *Ap2s1^+/del17^*. (*C*) Analysis of the DNA sequence of the variants in exon 2 revealed that an in‐frame cryptic donor splice site located at codon 35 that is not normally utilized has been used. (*D*) Multiple protein sequence alignment of residues within exon 2 and 3 of *Ap2s1* revealed that use of the cryptic donor splice site resulted in an in‐frame loss of 17 evolutionarily conserved amino acids, thereby indicating that they likely have important structure‐function roles in AP2σ. (*E*) Three‐dimensional modeling revealed that the 17 amino acids that are lost because of the donor splice site mutation would lead to a loss of part of the α1 helix, the α1‐β3 loop, and β3 strand (shown in green).

**Figure 5:**
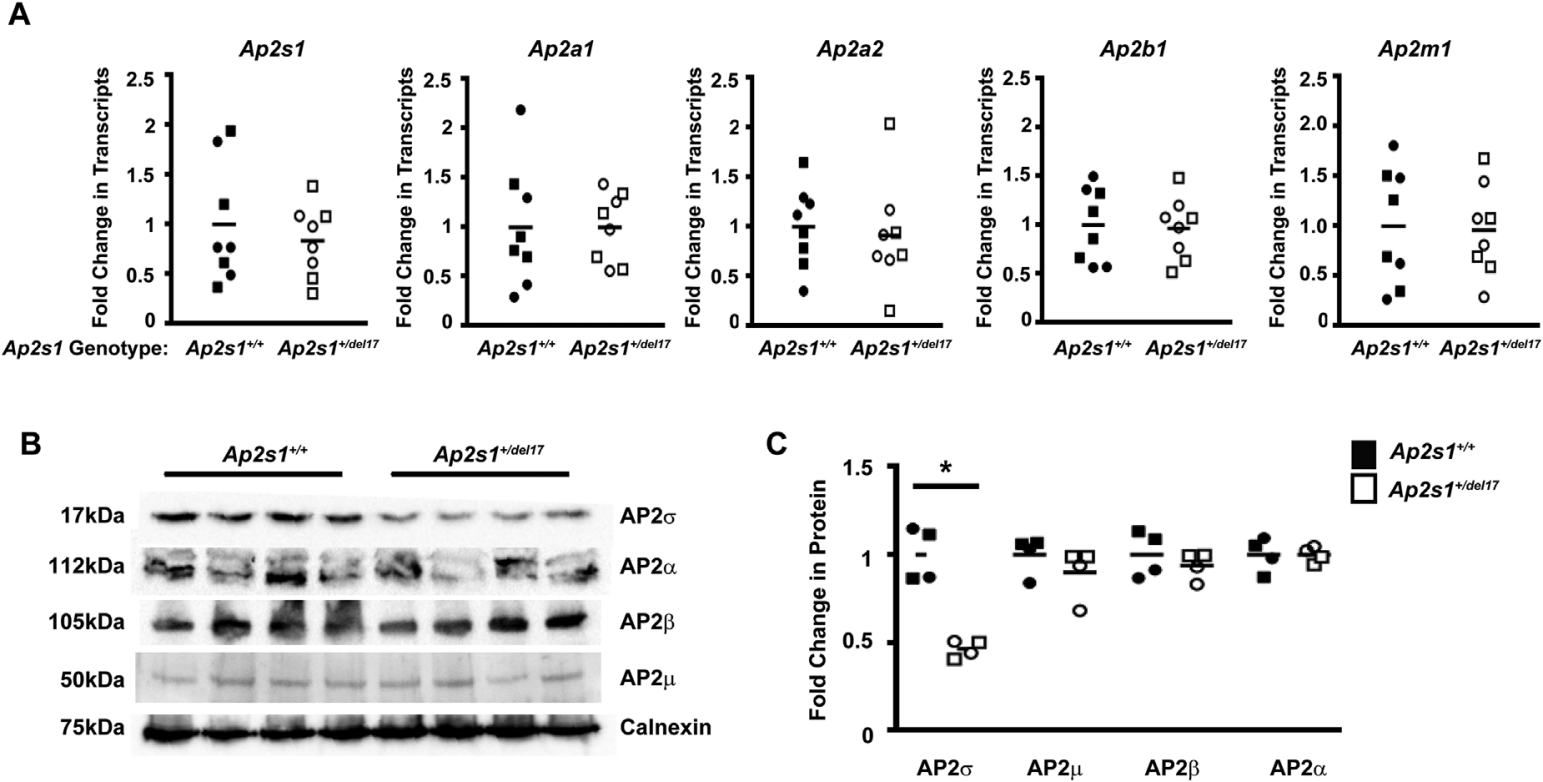
Effects of *Ap2s1* intron 2 donor splice site on transcription and translation of AP2 subunits. Assessment of transcription and translation of genes *Ap2s1*, *Ap2a1*, *Ap2a2*, *Ap2b1*, and *Ap2m1* encoding the subunits AP2σ, AP2α1, AP2α2, AP2β, and AP2μ, respectively, of the AP2 heterotetrameric complex in wild‐type (*Ap2s1^+/+^*) mice and heterozygous mutant mice with the *Ap2s1* intron 2 donor splice site mutation that results in loss of 17 amino acids (*Ap2s1* ^+/del17^). (*A*) Quantitative RT‐PCR (qRT‐PCR) analysis of the *Ap2s1*, *Ap2a1*, *Ap2a2*, *Ap2b1,* and *Ap2m1* transcripts using total RNA from kidneys of *Ap2s1^+/+^* and *Ap2s1* ^+/del17^ male and female mice. All data were normalized to the geometric mean of three housekeeper genes (*Ccnd1*, *Gapdh*, *Pgk1*) and expressed as a fold‐change compared with wild‐type (*Ap2s1^+/+^*) mice. Mean values for the respective groups are indicated by horizontal solid lines. Each point represents an individual mouse (males = squares; females = circles). There were no significant differences in the *Ap2s1, Ap2a1*, *Ap2a2*, *Ap2b1,* and *Ap2m1* transcript levels between *Ap2s1* ^+/+^ and *Ap2s1* ^+/del17^ mice or between sexes. (*B*) Assessment of AP2 subunits (α, β, μ, σ) by Western blot analysis, using kidney lysates from *Ap2s1* ^+/+^ and *Ap2s1* ^+/del17^ mice. Calnexin was used as the housekeeping protein and loading control. Each lane represents 1 mouse, and 2 male and 2 female mice were used for each of the *Ap2s1* ^+/+^ and *Ap2s1* ^+/del17^ mice. (*C*) Densitometry analysis of the expression levels of AP2 subunits (α, β, μ, σ). Data are from a total of 4 mice (2 males and 2 females) in each group. Data from males and females were combined as the transcript levels were similar in both sexes. Mean values for the respective groups are indicated by horizontal solid lines. Each point represents an individual mouse (males = squares; females = circles). AP2σ was significantly (*p < 0.05) reduced by ∼50% in *Ap2s1^+^* ^/del17^ mice, consistent with AP2σ haplosufficiency, whereas other subunits were unaffected.

Three intronic *Ap2s1* variants were identified and these comprised: a T‐to‐C transition at position −22 (IVS2‐22T>C) in intron 1 (Fig. 1); a T‐to‐C transition at position +2 (IVS2+2T>C) in intron 2, which disrupted the consensus donor splice site gtgac to gcgac (Figs. 1 and 4); and a C‐to‐T transition at position −17 (IVS5‐17C>T) in intron 4 (Fig. 1). The effects of these intronic variants were assessed using the Alternative Splicing Site Predictor, and this revealed that IVS2‐22T>C and IVS5‐17C>T would not result in splicing defects. However, IVS2+2T>C, which altered the invariant *gt* dinucleotide of donor splice sites to *gc* and predicted a loss of a donor splice site at the 5’ end of intron 2 of *Ap2s1*, could result in exon skipping, activation of a cryptic splice site within exon 2, or intron retention. These abnormalities could lead to a truncated AP2σ protein and yield a mouse with a deleted *Ap2s1* allele (knockout mouse model). The mouse harboring this IVS2+2T>C variant was therefore rederived to determine the effect on splicing and on calcium metabolism. Heterozygous mice (MGI designation: C3H.B6J‐*Ap2s1*<m1H>/H) with the donor splice site variant were viable, and the effects of the IVS2+2 donor splice site variant on splicing of the *Ap2s1* gene were studied using RT‐PCR with primers located in exons 1–3 (Fig. 1, Supplemental Table S1) and utilizing total RNA obtained from a wild‐type mouse and a mouse that was heterozygous for the intron 2 variant. This revealed that the intron 2 variant resulted in two PCR products of 267 bp and 216 bp compared with the one product of 267 bp from the wild‐type sample (Fig. 4 *A*). DNA sequence analysis of these RT‐PCR products revealed that the variant altering the donor splice site led to a loss of 51 nucleotides from exon 2 (Fig. 4 *B*, *C*), with a resulting in‐frame deletion of 17 evolutionarily conserved amino acids involving codons 35–51 (Fig. 4 *D*). Thus, the ENU‐induced mutation (T>C at IVS2+2), which resulted in disruption of the *gt* invariant dinucleotide of donor splice sites to *gc*, resulted in utilization of an alternate cryptic donor splice site (GTGCAC) within exon 2 that caused an in‐frame deletion of 17 (del17) amino acids that form part of the α1 helix, α1‐β3 loop, and the whole of the β3 strand of AP2σ (Fig. 4 *E*). The effects of this mutation, which is designated del17, on *Ap2s1* transcription and translation were studied using 4 kidneys obtained from heterozygous mutant mice (*Ap2s1* ^+/del17^) (Fig. 5). qRT‐PCR analysis revealed no differences in the mRNA expression levels of *Ap2s1* between wild‐type (*Ap2s1* ^+/+^) and mutant (*Ap2s1* ^+/del17^) mice, and between males and females (Fig. 5 *A*), which also did not have significant differences in the mRNA expression levels of the other genes encoding the AP2 subunits (α, β, and μ) (Fig. 5 *A*). Western blot analysis revealed that the *Ap2s1* ^+/del17^ mice expressed only one protein product, which corresponded to the full‐length 17kDa AP2σ protein (Fig. 5 *B*). However, expression of AP2σ was reduced in the mutant *Ap2s1* ^+/del17^ mice (fold‐change of 0.30 ± 0.01 for *Ap2s1* ^+/del17^ mice compared with 1 ± 0.24 for *Ap2s1* ^+/+^ mice, *p* < 0.05), consistent with AP2σ haplosufficiency and suggesting that the mutant AP2σ protein may be degraded or retained within the protein synthesis pathway (Fig. 5 *C*). However, this difference in AP2σ expression levels had no effect on the expression of the AP2α, AP2β, or AP2 μ subunits (Fig. 5 *B*, *C*). The phenotypic effects of this AP2σ haplosufficiency, due to the ENU‐induced donor splice site mutation of intron 2, on calcium metabolism were further investigated in the mutant mice.

### Phenotype studies of mutant mice with *Ap2s1* donor splice site mutation of intron 2

**Figure 6:**
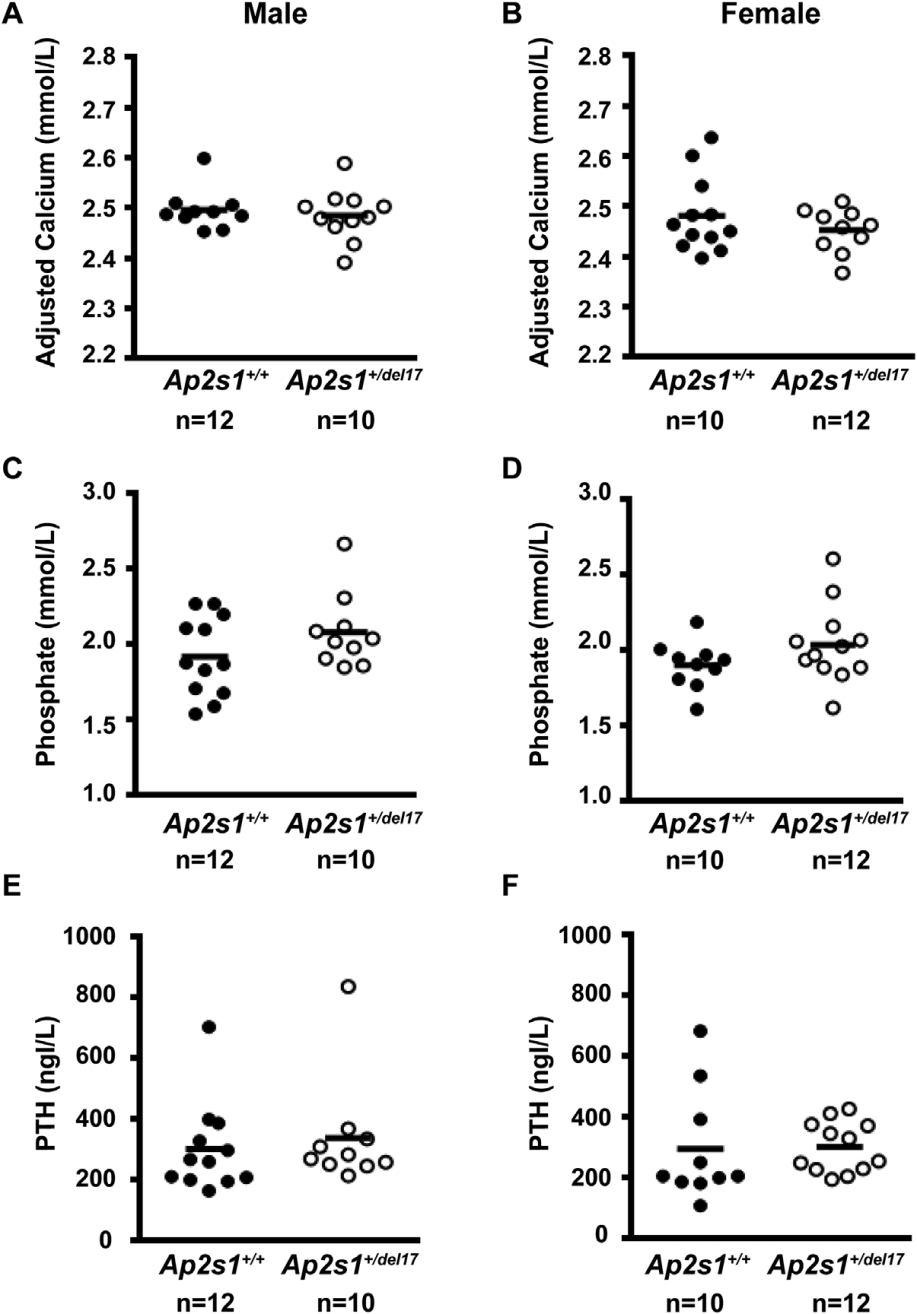
Plasma calcium, phosphate, and PTH studies in *Ap2s1^+/del17^* mice. (*A*, *B*) Plasma‐adjusted calcium, (*C*, *D*) plasma phosphate, and (*E*, *F*) plasma PTH concentrations in male and female wild‐type (*Ap2s1* ^+/+^) and heterozygous mutant (*Ap2s1^+/del17^*) mice. Each point represents an individual mouse. Mean values for the respective groups are indicated by horizontal solid lines. *Ap2s1^+/+^* and *Ap2s1^+/del17^* mice had similar plasma calcium, phosphate, and PTH concentrations, and these were also similar between the two sexes.

Analysis of offspring from crosses of heterozygous mutant (*Ap2s1^+/del17^*) mice with C3H/HeH *Ap2s1* ^+/+^ mice revealed the proportion of *Ap2s1* ^+/del17^ mice was as expected for a Mendelian pattern of inheritance (51.97% *Ap2s1^+/+^* versus 48.03% *Ap2s1^+/del17^* [for 304 pups]). *Ap2s1^+/del17^* mice were fertile, grew at similar rates as their *Ap2s1^+/+^* littermates, had similar body weights, and appeared morphologically normal. To determine the effects of the AP2σ haplosufficiency, resulting from the intron 2 donor splice site mutation of intron 2, on calcium and electrolyte homeostasis in vivo, plasma and urine samples from *Ap2s1* ^+/del17^ and *Ap2s1* ^+/+^ adult mice, aged 10 to 12 weeks, were analyzed. This revealed no significant differences between *Ap2s1* ^+/del17^ and *Ap2s1^+/+^* mice in plasma concentrations of albumin‐adjusted calcium, phosphate, PTH, 1,25‐dihyroxyvitamin D, magnesium, creatinine, urea, sodium, potassium, or alkaline phosphatase activity (Fig. 6, Table 1). Furthermore, there were no significant differences between *Ap2s1^+/+^* and *Ap2s1^+/del17^* mice in the urinary excretion of calcium, phosphate, sodium, or potassium (Table 2). These studies indicate *Ap2s1I* ^+/del17^ mice are not a model for FHH3 and that mechanisms other than haplosufficiency of AP2σ are responsible for causing FHH3.

**Table 1:** Plasma Biochemical Studies of *Ap2s1^+/del17^* Mice

	Male		Female	
	*Ap2s1^+/+^*	*Ap2s1* ^+/del17^	*Ap2s1^+/+^*	*Ap2s1* ^+/del17^
	*n* = 12	*n* = 10	*n* = 10	*n* = 12
Sodium (mmol/L)	150.75 ± 0.30	151.20 ± 0.20	148.60 ± 0.54	148.83 ± 0.37
Potassium (mmol/L)	5.46 ± 0.30	5.25 ± 0.11	4.73 ± 0.07	4.71 ± 0.06
Urea (mmol/L)	12.24 ± 0.42	13.07 ± 0.39	8.58 ± 0.48	8.51 ± 0.21
Creatinine (µmol/L)	15.64 ± 0.48	16.22 ± 0.69	14.09 ± 0.66	14.42 ± 0.39
Calcium (mmol/L)^a^	2.36 ± 0.02	2.34 ± 0.02	2.42 ± 0.01	2.41 ± 0.02
Magnesium (mmol/L)	0.79 ± 0.03	0.74 ± 0.02	0.79 ± 0.02	0.81 ± 0.02
Phosphate (mmol/L)	1.92 ± 0.76	1.90 ± 0.05	2.09 ± 0.08	2.04 ± 0.07
ALP (U/L)	101.33 ± 3.75	100.20 ± 3.94	146.10 ± 4.73	147.00 ± 6.57
PTH (ng/L)	302.98 ± 42.53	338.47 ± 57.28	296.26 ± 58.24	303.25 ± 24.13
1,25D (pmol/L)	68.77 ± 3.82	59.97 ± 4.25	88.67 ± 6.45	85.87 ± 6.78

Plasma biochemical analysis was performed on 10‐ to 12‐week‐old *Ap2s1* ^+/del17^ and *Ap2s1* ^+/+^ mice. All values are expressed as mean ± SEM.

ALP = alkaline phosphatase activity; PTH = parathyroid hormone; 1,25D = 1,25‐dihydroxyvitamin D.

^a^ Plasma calcium concentrations were adjusted for the plasma albumin concentration.

**Table 2:** Urine Biochemical Studies of *Ap2s1^+/del17^* Mice

	Male		Female	
	*Ap2s1^+/+^*	*Ap2s1* ^+/del17^	*Ap2s1^+/+^*	*Ap2s1* ^+/del17^
	*n* = 12	*n* = 10	*n* = 10	*n* = 12
24‐hour calcium	2.09 ± 0.15	2.44 ± 0.26	3.29 ± 0.32	4.42 ± 0.25
Calcium/creatinine	0.34 ± 0.026	0.36 ± 0.034	0.44 ± 0.032	0.51 ± 0.04
Fractional excretion calcium	0.002 ± 0.0001	0.003 ± 0.0003	0.003 ± 0.0002	0.003 ± 0.0002
Fractional excretion phosphate	0.04 ± 0.006	0.04 ± 0.010	0.02 ± 0.009	0.02 ± 0.006
Fractional excretion sodium	0.01 ± 0.0003	0.01 ± 0.0004	0.01 ± 0.0003	0.01 ± 0.0003
Fractional excretion potassium	0.29 ± 0.01	0.29 ± 0.01	0.27 ± 0.01	0.28 ± 0.01

Urine biochemical analysis was performed on 10‐ to 12‐week‐old *Ap2s1* ^+/del17^ and *Ap2s1* ^+/+^ mice, in metabolic cages, using urine samples collected over a 24‐hour period. Urinary calcium excretion values are shown as µmol/24 hours. All values are expressed as mean ± SEM.

Initial matings of heterozygous (*Ap2s1^+/del17^*) intercrosses did not yield any homozygote mutant (*Ap2s1^del17^* ^*/del*^ *^17^*) mice. For example, assessment of 25 live offspring from heterozygous intercrosses revealed that 9 were wild‐type (*Ap2s1* ^+/+^) mice, 16 were heterozygous (*Ap2s1* ^+/del17^) mice, and 0 were homozygous (*Ap2s1* ^del17/del17^) mice, which deviated significantly from that for a Mendelian inheritance (chi‐square test, *p* = 0.017, Table 3). We therefore performed timed matings and analyzed the genotype distribution at embryonic days 12.5 (E12.5), E9.5, and E3.5 (Table 3) to define the window of lethality. Analysis of 50 embryos at 12.5 days (E12.5), which is mid‐gestation, revealed a significant deviation from the expected ratio for a Mendelian inheritance (expected for wild‐type (*Ap2s1^+/+^*): heterozygous mutant (*Ap2s1^+/del17^*): homozygous mutants (*Ap2s1^del17^* ^*/del*^ *^17^*) = 12:26:12 versus observed = 15:34:1, *p* < 0.0001) (Table 3). Furthermore, the resorption rate at E12.5 was 19% in pregnancies from the *Ap2s1^+/del17^* intercrosses, which is significantly (chi‐square test, *p* = 0.012) higher than the reported 10.22% rate of spontaneous resorption in wild‐type mice,^37^ indicating that most *Ap2s1^del17^* ^*/del*^ *^17^* mice are dying before this stage. Analysis of 60 embryos at an earlier stage, E9.5, revealed a significant deviation from the expected ratio, with no *Ap2s1^del17^* ^*/del*^ *^17^* embryos (Table 3) and with a significantly high rate of resorption of 40% (chi‐square test, *p* < 0.001). However, the expected Mendelian inheritance ratio was observed among 42 blastocysts (at ∼E3.5) (Table 3), indicating that AP2σ may play a critical role in mouse embryonic development between E3.5 and E9.5. Thus, this observed embryonic lethality of homozygous mutant (*Ap2s1^del17^* ^*/del*^ *^17^*) mice indicates that AP2σ has an important role in embryonic development.

**Table 3:** Progeny From *Ap2s1^+/del17^* × *Ap2s1^+/del17^* Intercrosses

		Genotype				
		*Ap2s1^+/+^*	*Ap2s1* ^+/del17^	*Ap2s1* ^del17/del17^	*Total*	*p* Value
Stage						
Live birth	Expected^a^	6	13	6	25	0.017
	Observed	9	16	0		
E12.5	Expected^a^	12	26	12	50	0.0001
	Observed	15	34	1		
E9.5	Expected^a^	15	30	15	60	0.0001
	Observed	27	33	0		
Blastocyst^a^ (∼E3.5)	Expected^a^	10	22	10	42	0.31
	Observed	10	26	6		

E = embryonic day.

Statistical analyses were performed by chi‐square analysis comparing expected to observed numbers for each litter.

^a^ Expected ratio from heterozygous intercrosses for Mendelian inheritance of wild‐type (*Ap2s1* ^+/+^): heterozygotes (*Ap2s1* ^+/del17^): homozygotes (*Ap2s1* ^del17/del17^) = 1:2:1.

## Discussion

Our studies have established a mouse with an *Ap2s1* donor splice site mutation of intron 2 (Fig. 1) that results in a loss of 17 amino acids (del17), which form parts of the α1 helix, α1‐β3 loop, and the whole of the β3 strand of the AP2σ subunit (Fig. 4). This mutation leads to haplosufficiency in heterozygous mutant mice (*Ap2s1^++/del17^*) (Fig. 5), which appear phenotypically normal and are viable, in contrast to homozygous mutant mice (*Ap2s1^del17^* ^*/del*^ *^17^*), which have embryonic lethality after the blastocyst stage (E3.5) (Table 3). Our findings in mice with mutant AP2σ, which is involved in recognition of cargo proteins with dileucine motifs for endocytosis, are similar to those reported in mice with deletion of AP2μ, which is the other small subunit of AP2 and is involved in recognition of cargo proteins with tyrosine‐based motifs for endocytosis.^38^ Thus, heterozygous mutant AP2μ mice (*Ap2μ^+/−^*) have an apparently normal phenotype and are viable, whereas homozygous mutant mice (*Ap2μ^+/−^*) were embryonically lethal and die before E3.5, indicating an essential role for AP2μ in early embryonic development and in survival of embryonic cells.^39^ Our results, which demonstrate embryonic lethality of *Ap2s1^del17^* ^*/del*^ *^17^* mice between E3.5 and E9.5, indicate that AP2σ also has critical roles in early embryonic development stages, which include patterning of the embryo and organogenesis. Thus, AP2σ may have roles in patterning of the embryo, with development of the anterior‐posterior axis and dorsal‐ventral axis, which occur at E6.5, and left‐right asymmetry and heart tube development, which occur at E8.5;^40^ and organogenesis of the thyroid (commencing from E8.5), liver (commencing from E9.5), gut (commencing before E9.5), lungs (commencing from E9.5), pancreas (commencing from E9.5), and neural tube closure commencing at E8.25.^41^ However, a major distinction between AP2σ and AP2μ null mice is that the former survive beyond E3.5, whereas the latter do not. This indicates that AP2σ and AP2μ have functionally distinct roles in development that may be due to recognition of different developmental endocytic cargos.^42^ At present, the exact cargos of AP2σ and AP2μ during embryonic development are unknown, and further investigation of this could highlight the differences in stages of embryonic lethality. The early embryonic lethality of these AP2σ and AP2μ null mice differs markedly to the situation of mice null for AP2β, which is one of the larger subunits of AP2. Thus, AP2β null mice survive to birth but have a high perinatal mortality because of developmental defects, including cleft palates.^43^ The survival of AP2β null mice has been attributed to the partial functional redundancy of the β‐subunit by its paralog in the AP1 complex, which is involved in trans‐Golgi‐network trafficking.^43^ The β‐subunits of AP1 and AP2 are 84% identical at the amino acid level,^43^ thereby allowing some interchangeability between the subunits. However, other adaptor protein subunits are less similar, including the μ and σ subunits, which cannot substitute for each other,^44^ and this may help to explain the observed early embryonic lethality in mice null for AP2μ and AP2σ.

In contrast to the early embryonic lethality of homozygous mutant *Ap2s1* mice (*Ap2s1^del17^* ^*/del*^ *^17^*), heterozygous mutant *Ap2s1* mice (*Ap2s1^+/del17^*) were viable and appeared normal, and this is similar to the findings in heterozygous *Ap2μ^+/−^* and *Ap2β^+/−^* mice.^39^, ^43^ However, the *Ap2s1^+/del17^* did not have the plasma and urine biochemical abnormalities associated with FHH3, and these included an absence of hypercalcemia and hypocalciuria (Fig. 6, Tables 1 and 2). These findings indicate that AP2σ haplosufficiency (Fig. 5), which is observed in these heterozygous mutant mice, is not responsible for FHH3. This is consistent with our studies in FHH3 patients that reported that FHH3‐associated AP2σ mutations, which are all missense mutations of the Arg15 residue that binds to the dileucine motifs of cargo proteins, exert a dominant‐negative effect on the function of the AP2 complex.^12^ In this context, our findings related to the two missense AP2σ variants Tyr20Asn and Ile123Asn (Fig. 1) help to illustrate some important points. These two missense AP2σ variants (Figs. 1 and 2), which involved evolutionarily conserved residues and were predicted to be damaging and disease‐causing, had not been previously reported in the ExAc or NHLBI‐ESP databases, that contain data from >66,000 individuals,^36^ thereby further supporting the likelihood that they may be disease‐causing mutations. However, these missense ENU‐induced variants, Tyr20Asn and Ile123Asn, were found not to alter CaSR‐mediated signaling by in vitro assays (Fig. 3), and the molecular mechanism for this tolerance of the altered residues at codons 20 and 123 is likely owing to the position of the respective residues within the AP2σ protein. Thus, Tyr20 lies within a region distal to the region of the AP2σ that interacts with dileucine motifs and is not close to regions of the protein known to be involved in interactions with other AP2 complex proteins (Fig. 2). Similarly, Ile123 lies within a loop structure close to the binding regions with other AP2 complex proteins, but the mutation is not predicted to disrupt any structures within AP2σ or to generate steric hindrance within the structure (Fig. 2). Moreover, it seems that there may be a low frequency of *Ap2s1* mutations as only five missense mutations and no splice or nonsense mutations have been reported in the ExAc and NHLBI‐ESP databases.^36^ Indeed, the observed frequencies of missense and nonsense *AP2S1* variants is significantly lower than that expected from the ExAc database^36^ (observed *Ap2s1* variants versus ExAc gene variants: missense = 0.008% versus 0.1%; nonsense = 0% versus 0.01%, chi‐square *p* < 0.0001). This suggests that mutations that are predicted to disrupt the structure of AP2σ, or its functions such as cargo recognition, are not tolerated. Thus, our studies highlight the limitations of bioinformatics and protein prediction software, which may be inaccurate,^45^ and the importance of acquiring data from multiple sources, including modeling of mutations to predict possible outcomes of missense changes and in vitro functional characterization to assess known roles of the protein.

In summary, we have identified five ENU‐induced *Ap2s1* variants and demonstrated that one of these resulted in loss of a donor splice site in intron 2 and was associated with a loss of 17 amino acids (del17) that form part of the α1 helix, α1‐β3 loop, and β3 strand of the AP2σ subunit. In vivo investigations of heterozygous *Ap2s1^+/del17^* mice revealed them to have AP2σ haplosufficiency that was associated with a normal appearance, normal plasma, and urine calcium concentrations and normal plasma PTH concentrations. However, homozygous *Ap2s1^del17^* ^*/del*^ *^17^* mice had embryonic lethality commencing after E3.5, indicating an important role for AP2σ in embryonic patterning and organogenesis.

## Disclosures

All authors state that they have no conflicts of interest.

## Supporting information

Supporting Table S1.
