# Supplementary material for: N‐ethyl‐N‐nitrosourea–Induced Adaptor Protein 2 Sigma Subunit 1 (Ap2s1) Mutations Establish Ap2s1 Loss‐of‐Function Mice
Source: JBMR Plus. 2017 May 2;1(1):3–15. doi: 10.1002/jbm4.10001 (PMC5824975; doi:10.1002/jbm4.10001)
Supplement: Supplementary file 1 — Supporting Table S1. [file JBM4-1-3-s001.docx]

|  |  | **Primer sequence** | **Annealing temperature (°C)** |
| --- | --- | --- | --- |
| **Primers for genotyping of *Ap2s1^+/del17^*mouse** | Forward | TCTCCCATAGAATGTCTCGT | 60 |
|  | Reverse | CAACGAGCCACATCCACAAC | 60 |
| **Primers for RT-PCR of *Ap2s1*** | Forward | ATGATCCGATTCATCCTTAT | 60 |
|  | Reverse | TTCTACGAAGTTGTGGATGGCC | 60 |
| **Primers for sequencing pCR-BluntII TOPO vector** | Forward | GTAAAACGACGGCCAG | N/A |
|  | Reverse | CAGGAAACAGCTATGAC | N/A |

**Supplementary Table 1 Primers used in genotyping and RT-PCR studies**

Sequences for primers used for: mouse genotyping; RT-PCR of cDNA from *Ap2s1* mice; and, for sequencing the pCR-BluntII-TOPO. Primers for qRT-PCR and the Lightscanner are gene-specific and details are available from QuantiTect and BioFire Diagnostics, respectively.
